# Longitudinal ^18^F-VUIIS1008 PET imaging in a rat model of rheumatoid arthritis

**DOI:** 10.3389/fchem.2022.1064518

**Published:** 2022-12-23

**Authors:** Xinhui Su, Liangliang Wang, Rongshui Yang, Zhide Guo

**Affiliations:** ^1^ Department of Nuclear Medicine, School of Medicine, The First Affiliated Hospital, Zhejiang University, Hangzhou, China; ^2^ The School of Clinical Medicine, Fujian Medical University, Fuzhou, China; ^3^ Department of Nuclear Medicine, Zhongshan Hospital Xiamen University, Xiamen, China; ^4^ Department of Nuclear Medicine, Linyi People’s Hospital, Linyi, China; ^5^ Center for Molecular Imaging and Translational Medicine, Xiamen University, Xiamen, China

**Keywords:** 18F-VUIIS1008, PET, rheumatoid arthritis, TSPO 18 kDa, macrophages

## Abstract

Macrophages have crucial roles in the pathogenesis of rheumatoid arthritis (RA). We aimed to elucidate the temporal profile of macrophage infiltration in synovitis in RA rat models using PET (positron emission tomography) imaging based a new generation of TSPO (Translocator protein, 18 kDa)-PET ligand, ^18^F-VUIIS1008 {2-[5,7-Diethyl-2-{4-[2-(^18^F)fluoroethoxy]phenyl}pyrazolo(1,5-a)pyri-midin-3-yl]-N, N-diethylacetamide}. *In vitro* and *in vivo* studies were conducted using RAW264.7 macrophage cells and a rat model of RA induced by Complete Freund’s Adjuvant (CFA). Our results showed ^18^F-VUIIS1008 showed excellent stability *in vitro* and binding specificity to RAW264.7 cells, and rapid accumulation in the left inflammatory ankles. PET studies revealed that ^18^F-VUIIS1008 could clearly identify the left inflammatory ankles with good contrast at 30–120 min post-injection. The uptake of ^18^F-VUIIS1008 of left inflammatory ankles was a wiggle trace with two peaks on day 7 and 29, and then, the highest peak uptake was seen on day 29 (3.00% ± 0.08%ID/g) at 60 min after injection. Tracer uptakes could be inhibited by PK11195 or VUIIS1008. Immunohistochemistry and immunofluorescence tests showed that elevated TSPO expression and infiltrated macrophages were found in the left inflammation ankles. ^18^F-VUIIS1008 as a novel PET imaging agent showed great potential to identify temporal profile of macrophage infiltration in synovitis in RA, and deliver accurate non-invasive diagnosis and real-time monitoring of RA development.

## 1 Introduction

Rheumatoid arthritis (RA) is a chronic systemic autoimmune inflammatory disease primarily characterized by chronic joint inflammation, cartilage destruction and bone erosion, leading to severe progressive joint damage, functional disability, morbidity, and increased mortality (Calabrò et al., 2016). RA is approximately three-times more common in women than in men and affects 0.5%–1.0% of the world’s population (De Cata et al., 2014). The main objective of RA treatment is to stop inflammation, relieve symptoms, prevent joint and organ damage, improve physical function and reduce long-term complications (Jalil et al., 2016). The common treatment method is anti-inflammation early in the disease course as soon as the diagnostic has been established, suggesting that the early diagnosis is a key for the therapy and prognosis of RA.

Although the pathogenesis of RA is not yet completely understood, it is considered as a complex, multi-factorial etiology, including genetic sensitivity, and environment factors and autoimmune responses, which collectively trigger the onset and persistence of inflammatory circumstance (Firestein, 2003). Mounting data of evidence have shown that the degree of macrophage infiltration into the synovium is correlated with the degree of bone erosion in the affected joints in RA (Soler Palacios et al., 2015; Udalova et al., 2016), since pro-inflammatory cytokines from activated macrophage, such as tumor necrosis factor-α (TNF-α), interleukin-1 (IL-1), and interleukin-6 (IL-6), contribute to synovial inflammation in early stages of RA (Arthur and Ley, 2013). The abundance of synovial tissue macrophages is an early RA hallmark (Kurowska-Stolarska and Alivernini, 2022). Therefore, the imaging using a specific probe targeted activated macrophage possibly enable an earlier detection of RA. Recently, specific ligands targeting macrophage receptors such as CD20 receptor, interleukin-1 (IL-1) receptor, *etc.*, have been investigated in the patients with RA using ^99m^Tc-anti-CD20, ^123^I-IL-1ra and ^124^I-anti-CD20 illustrating the interest for molecular imaging in this type of pathology (Barrera et al., 2000; Tran et al., 2011; Malviya et al., 2012). The drawbacks of probes with antibodies severely hamper their clinical applications due to their large size resulting in slow inflammation accumulation and slow clearance from the circulation (Su et al., 2015).

The translocator protein 18 kDa (TSPO), previously known as the peripheral-type benzodiazepine receptor (PBR), is located in the outer mitochondrial membrane, where involved in apoptosis, cell proliferation, anion transport, regulation of mitochondrial functions and immunomodulation (Papadopoulos et al., 2006). TSPO is a potential candidate for individualized approach to inflammation as its expression is enhanced in activated macrophage but it is low in the normal macrophage (Kanegawa et al., 2016). Thus, TSPO is regarded as a potential target for inflammatory diseases (Gatliff and CampanellaTSPO, 2016). It has been reported that positron emission tomography (PET) or single photon emission computed tomography (SPECT) study targeted TSPO probes, including ^11^C-(R)-PK11195, ^11^C-DPA-713, ^18^F-DPA-714 or ^99m^Tc-DTPA-CB86, can visualize RA (Gent et al., 2014a; Gent et al., 2014b; Liu et al., 2020). However, to our knowledge, few studies on TSPO imaging *in vivo* assessing the complete time course of joint inflammation during complete Freund’s adjuvant (CFA)-induced RA have been reported. Establishing such data may be important for the subsequent development of image-guided anti-inflammation interventions. Compared with ^11^C and ^99m^Tc, ^18^F may improve imaging of TSPO-expression and is more suitable for clinical application. In addition, a novel TSPO ligand (2-(5,7-diethyl-2-(4-(3-fluoro- 2-methylpropoxy)phenyl)pyrazolo[1,5-*a*]pyrimidin-3-yl)-*N,N*-diethylacetamide, VUI- IS1008), has been proved a 36-fold enhancement in binding affinity (*K*_*i*_ = 0.3 ± 0.14 nM) compared to DPA-714 (*K*_*i*_ = 10.9 ± 0.39 nM) (Tang et al., 2014). Furthermore, Kwon YD, et al., have reported that in a rat lipopolysaccharide (LPS)-induced neuroinflammation model, the uptake ratio of ^18^F-VUIIS1008 between the neuroinflammation ipsilateral and contralateral regions in the brain was 18% higher than that of ^18^F-DPA-714, suggesting that ^18^F-VUIIS1008 has better PET imaging tracer’s features for identifying neuroinflammation in brain than that of ^18^F-DPA-714 (Kwon et al., 2018). Accordingly, in the present study, we aimed to elucidate the potential role of longitudinal ^18^F-VUIIS1008 PET imaging in an experimental RA.

## 2 Materials and methods

### 2.1 General

VUIIS1008, a new TSPO agent, was presented by Professor Shoufa Han (College of Chemistry and Chemical Engineering, Xiamen University) according to the previous study by Kwon YD (Kwon et al., 2018). No-carrier-added ^18^F-fluoride was kindly provided by the First Affiliated Hospital of Xiamen University. Freund’s Adjuvant and anti-TSPO antibodies were purchased from Sigma-Aldrich Shanghai Trading Co Ltd. (Shanghai, China). Goat anti-mouse IgG antibody was from Santa Cruz Biotechnology Inc. (Santa Cruz, California, United States). WIZARD 2480 gamma counter from Perkin-Elmer Inc. (Waltham, MA, United States). CRC-25R Dose Calibrator from Capintec Inc. (Ramsey, New Jersey, United States). Mouse macrophage RAW264.7 cell lines were obtained from the Cell Culture Center of Institute of Basic Medical Sciences of Chinese Academy of Medical Sciences (Beijing, China). Male Wistar rats, aged 6–8 weeks (200–300 g), were purchased from the Experimental Animal Center of Xiamen University (Xiamen, China). Small animal PET/CT imaging studies were performed using a micro-PET/CT scanner (Inveon, Siemens Medical Solutions United States, Inc.).

### 2.2 Chemistry and radiochemistry

The synthesis of radiotracers 2-(5,7-diethyl-2-(4-(2-fluoroethoxy) phenyl) pyrazolo [1,5-a] pyrimidin-3-yl)-N, N-diethylacetamide (^18^F-VUIIS1008) were prepared from its corresponding tosylate precursors *via* manual synthesis according to previously reported procedures (Tang et al., 2013; Kwon et al., 2018). Briefly, aqueous ^18^F-fluoride (5–15 mCi; 0.2∼0.6 GBq) was eluted from the cartridge with a solution of Kryptofix K2.2.2 to form the complexation mixture. This complex was then reacted with appropriate tosylate precursor VUIIS1008 (4.0 mg) in dimethylsulfoxide (0.7 mL) at 100°C for 15min. The reaction crude was purified using semi-preparative HPLC (C18, Dynamax 250 × 10 mm; Varian), eluting with 10 mM NaH_2_PO_4_ buffer (pH 6.7) and methanol (30/70, v/v) at 3.0 mL/min. The product (^18^F-VUIIS1008) was collected directly into 140 ml of water (deionized), passed through a C-18 September-Pak Plus (Waters, Milford, MA, United States of America), and eluted with 200 proof ethanol (1.0 mL) then saline (0.9%) into a sterile vial.

### 2.3 Lipophilicity test of ^18^F-VUIIS1008

According to our previous report (Liu et al., 2020), the lipophilicity of ^18^F-VUIIS1008 was analysed by the n-octanol/water mixture containing 200 μL ^18^F-VUIIS1008 and 1 mL phosphate-buffered saline (pH = 7.4). The solution was centrifuged at 6,000 rpm for 5 min, and separated and then they were counted in a γ counter, respectively. The radioactivity were used to calculate the log *p* values. The lipophilicity of ^18^F-VUIIS1008 was determined as (cpm in organic phase)/ (cpm in water phase).

### 2.4 Stability studies

Based on our previous report (Liu et al., 2020), the stability of the complex containing 500 μL (3.7 MBq) ^18^F-VUIIS1008 and phosphate-buffered saline (PBS, pH = 7.4) or mouse serum was evaluated by performing the complex at 37°C for 30、60、120、and 240 min. The radioactivity of ^18^F-VUIIS1008 was measured at various time points by a HPLC.

### 2.5 Cell tests

The RAW264.7 cell lines were conducted cell uptake, and efflux tests in accordance with our previous study (Liu et al., 2020).

#### 2.5.1 Cell uptake tests

The RAW264.7 cell lines were cultured at 37°C for 15, 30, 60 and 120 min in the complex containing 0.5 mL serum-free DMEM medium and 7.4 × 10^–3^ MBq 100 μL ^18^F-VUIIS1008 with/without 10.0 μg unlabeled VUIIS1008, and then were lysed with 1 mL 1 M NaOH. The radioactivity of the lysates was measured at various time points by a γ counter.

#### 2.5.2 Cell efflux tests

The RAW264.7 cells were cultured at 37°C for 120, 135, 150 and 180, 240 min in the culture medium with 1.11 × 10^−2^ MBq 100 μL ^18^F-VUIIS1008 and then were lysed with 1 mL 1 M NaOH. The radioactivity of the lysates was measured at various time points by a γ counter.

### 2.6 Rat models with RA

The animal study protocol was carried out according to the principles outlines by the Institutional Animal Care and Use Committee of Zhongshan Hospital Xiamen University. The left inflammatory ankles were induced in male Wistar rats in accordance with our previous study (Liu et al., 2020). Briefly, 0.1 mL of Complete Freund’s Adjuvant (CFA) with Mycobacterium butyricum 1% suspension in mineral oil was injected into the left ankle of each rat (day 0). The severity of RA was monitored daily by two observers. The left inflammatory ankles were estimated by the number swollen joints. When the left inflammatory ankles grew to swell in two to three joints, the RA rats were subject to *in vivo* biodistribution and PET studies.

### 2.7 Biodistribution analysis

The biodistribution analysis were induced on day 3 after CFA injection. RA rats were administrated with ^18^F-VUIIS1008 (3.7 MBq, 100 μL) *via* tail vein. At 30, 60, and 120 min post-injection, the left inflammatory ankles and normal tissues of interest were removed and determined their radioactivity with a γ counter. For *in vivo* specificity study, RA rats were injected with ^18^F-VUIIS1008 and unlabeled PK11195 (500 μg), and biodistribution studies were performed at 60 min post-injection. The radioactivity ratios of the left inflammatory ankle to blood (LIA/B) and the left inflammatory ankle to muscle (LIA/M) were calculated. Biodistribution data are expressed as %ID/g values by dividing counts per gram per minute by the injected dose.

### 2.8 Micro-PET studies

PET imaging studies were performed using a micro-PET scanner (Siemens Medical Solutions United States, Inc.). Static PET imaging was performed at 30, 60, and 120 min post-injection of 3.7 MBq 100 μL ^18^F-VUIIS1008 *via* tail vein on day 3, 7, 12, 15, 19, 24, 29, 35, 38, and 45 after CFA injection. For blocking imaging, unlabeled PK11195 (500 μg) or VUIIS1008 (500 μg) was co-injected with ^18^F-VUIIS1008 (3.7 MBq 100 μ) on day seven. The RA rat were anesthetized with 2% isoflurane and positioned prone in micro-PET bed. Micro-PET images were reconstructed using an 3D OSEM scatter corrected reconstruction algorithm. Regions of interest (ROIs) were placed on the left inflammatory ankles. Micro-PET data are expressed as %ID/g values by dividing counts per gram per minute by the injected dose.

### 2.9 Histological studies

According to routine protocols, Hematoxylin and Eosin (HE staining), immunohistochemistry (IHC) tests and immunofluorescence staining were carried out in the tissues of left inflammatory ankles, contralateral normal ankles on day 3 after CFA injection. For HE tests, 5 𝜇m longitudinal sections were stained with hematoxylin and Eosin solution for 5 and 3 min at 25°C, respectively, and then analysed using an Olympus BX53 fluorescence microscope (Tokyo, Japan). For immunohistochemical analyses, the slices successively incubated with rabbit anti-rat TSPO antibodies (1:100, Abcam) and goat anti-rat secondary antibodies (1:1,000; Sigma) for 2 h at 25°C, and then analysed using an Olympus BX53 fluorescence microscope. For immunofluorescence staining, according to a standard protocol (Capaccione et al., 2020). The slides successively incubated with rabbit anti-rat TSPO antibodies (1:100, Abcam), anti-mouse CB68 antibodies (1:100, Abcam), and goat anti-rat FITC-IgG secondary antibodies (1:200; Sigma), goat anti-mouse TRITC-IgG secondary antibodies (1:200; Sigma), respectively, for 2 h at 25°C, and then stained using 200–300 μL 10 μg/mL of DAPI. After then, These slides were analysed using an Olympus BX53 fluorescence microscope.

### 2.10 Statistical analysis

The experimental data are presented as mean ± standard deviation. Statistical calculations were determined using the Student’s t*-*test and *p* < .05 was statistically significant.

## 3 Results and discussion

### 3.1 Radiosynthesis of ^18^F-VUIIS1008 and log P determination

**FIGURE 1:**
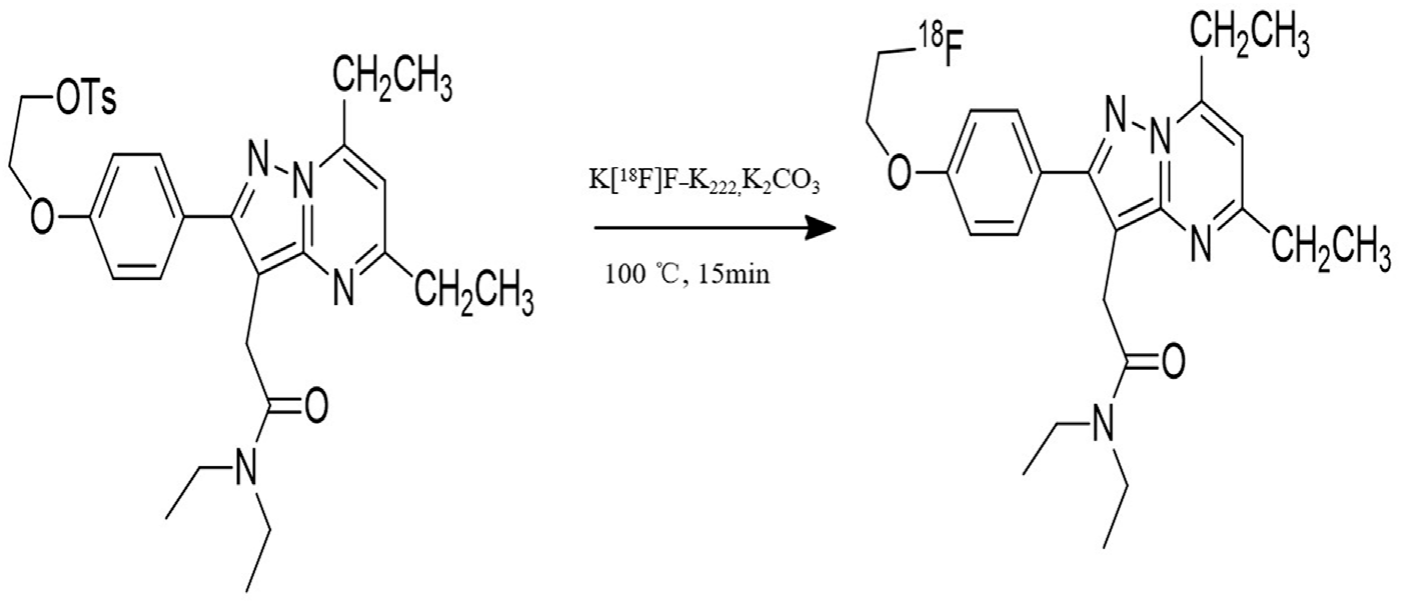
Synthetic scheme of ^18^F-VUIIS1008.

^18^F-VUIIS1008 was successfully radiosynthesized (Figure1). Under radio-HPLC conditions described above, ^18^F-VUIIS1008 displayed a retention time of 11.8 min. The radiochemical purity of the radiopharmaceutical exceeded 98.00%, and the specific activity of the purified ^18^F-VUIIS1008 was 1.52 × 10^8^ MBq/mmol. The lipid-water partition coefficient (log *P*) of ^18^F-VUIIS1008 is 1.58 ± 0.03, indicating ^18^F-VUIIS1008 is a fat-soluble compound.

### 3.2 Stability studies

**FIGURE 2:**
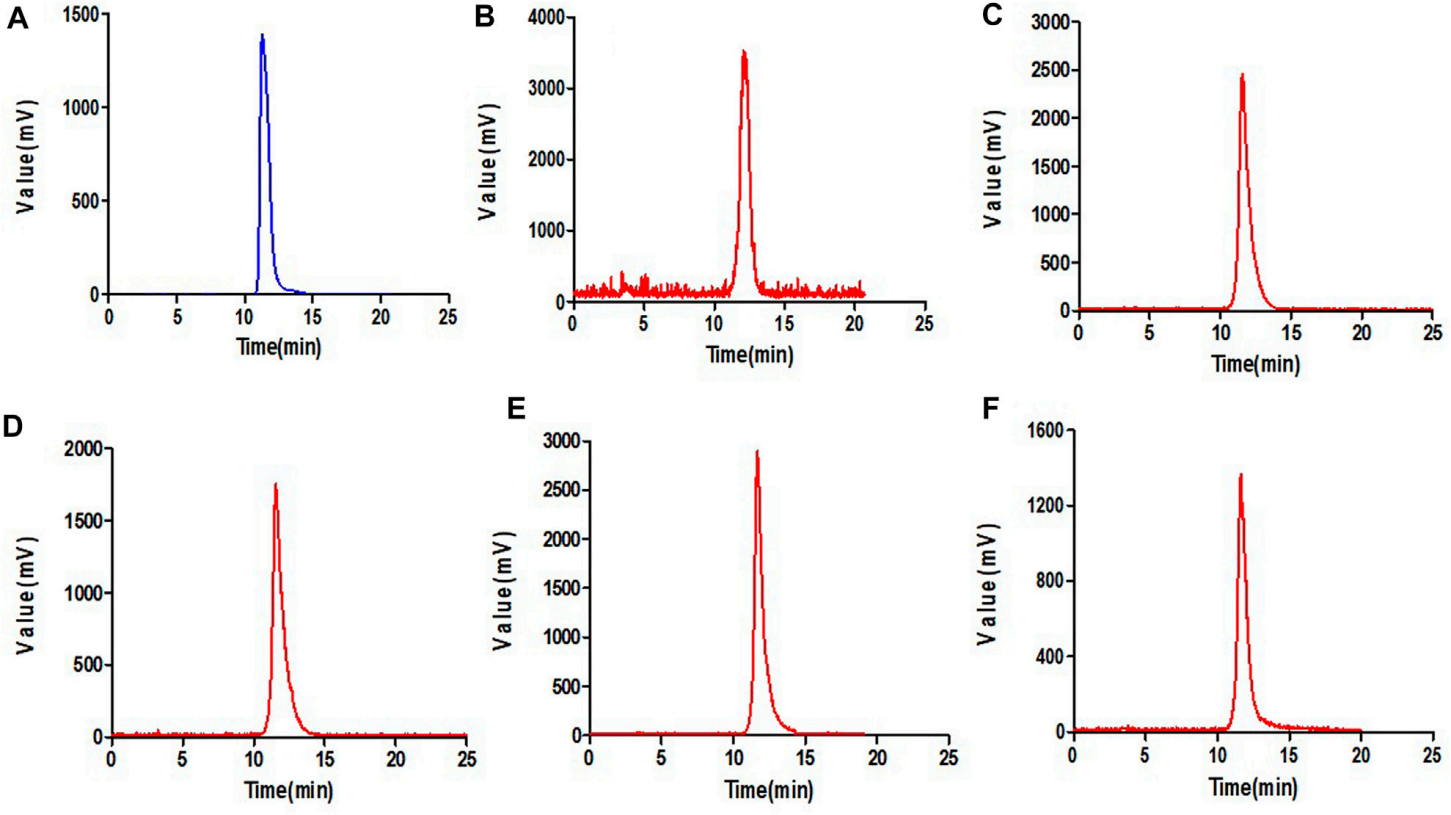
Prep-HPLC chromatogrm **(A)** and HPLC radiochromatograms of purified ^18^F-VUIIS1008 **(B)** and radiola-beled probe after 30 min **(C)**, 60 min **(D)**, 120 min **(E)** and 240 min **(F)** of incubation with PBS.

**FIGURE 3:**
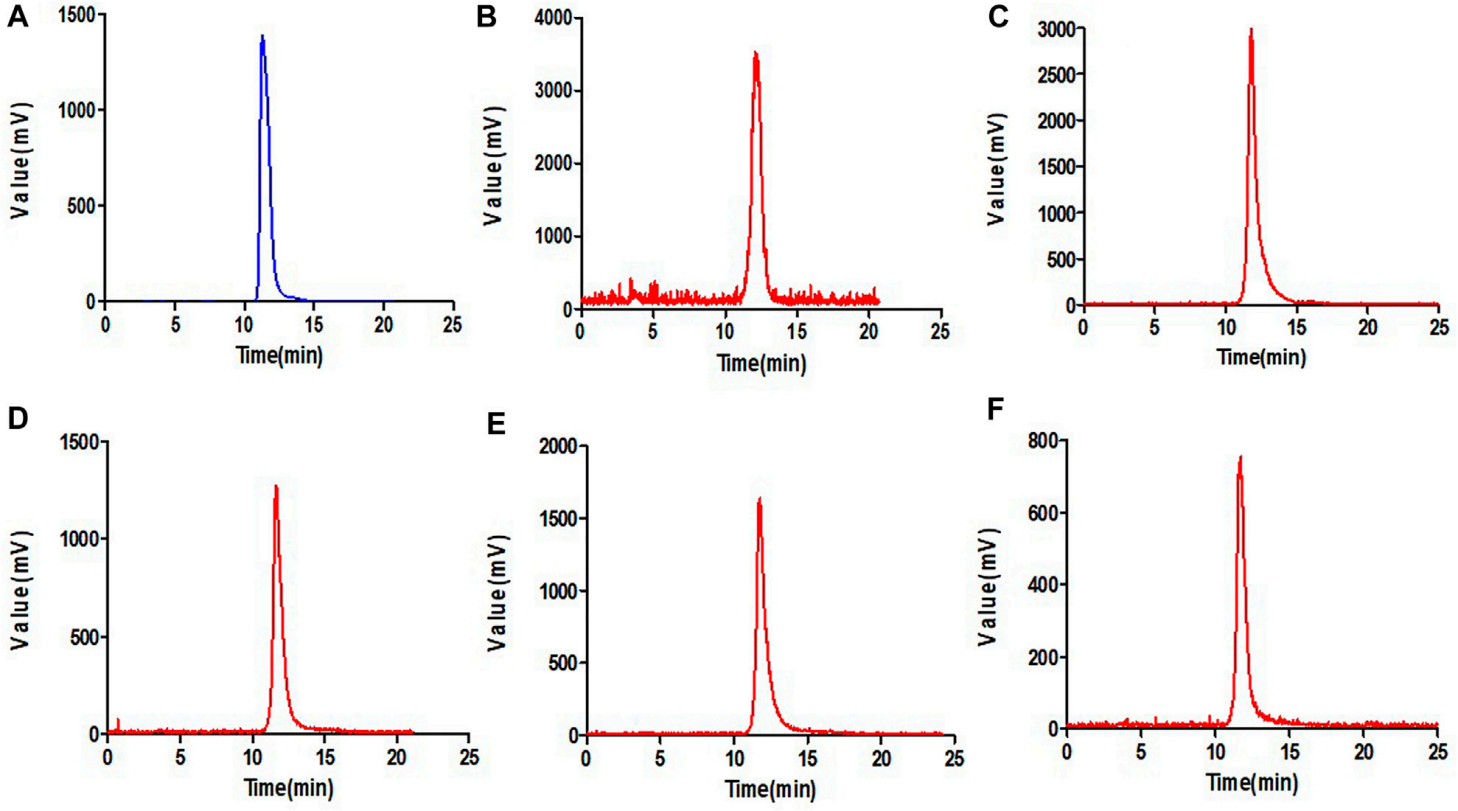
Prep-HPLC chromatogrm **(A)** and HPLC radiochromatograms of purified ^18^F-VUIIS1008 **(B)** and radiola-beled probe after 30 min **(C)**, 60 min **(D)**, 120 min **(E)** and 240 min **(F)** of incubation with mouse serum.

^18^F-VUIIS1008 displayed excellent stability in the PBS (Figure 2) or mouse serum (Figure 3). It showed that defluorination of ^18^F-VUIIS1008 was not obviously found, and the percentage of intact probes remained more than 90% during 30–240 min of incubation in the PBS or mouse serum.

### 3.3 Cell assays

**FIGURE 4:**
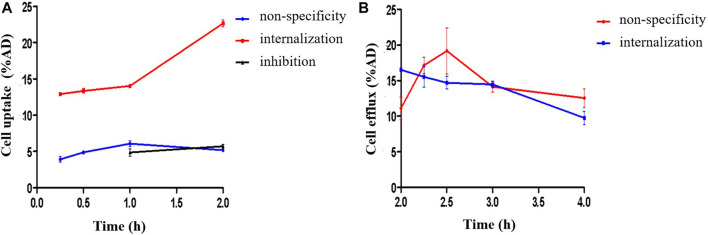
Uptake **(A)**, and efflux assay **(B)** of ^18^F-VUIIS1008 in RAW264.7 cells.

Cell uptake ratios of ^18^F-VUIIS1008 were shown in Figure 4A. The level of ^18^F-VUIIS1008 in RAW264.7 cells was 12.00 ± 0.10%, 13.00 ± 1.00%, 14.00 ± 0.30% and 23.00 ± 0.60% at 15, 30, 60, and 120 min, respectively. When the probe was incubated with large excesses of non-radioactive VUIIS1008, its uptake levels in RAW264.7cells was significantly inhibited (*p* <0.05) at all incubation time points. Moreover, cell efflux studies (Figure 4B) indicated ^18^F-VUIIS1008 has excellent cell retention in RAW264.7 cells, which ^18^F-VUIIS1008 efflux was 6.74% (reduction from 16.50 ± 0.002% to 9.76 ± 0.001% of total input radioactivity) from 120 min to 240 min incubation. In general, the results demonstrated that ^18^F-VUIIS1008 maintained high affinity to TSPO to further study *in vivo* TSPOtargeted imaging.

### 3.4 Biodistribution studies

**FIGURE 5:**
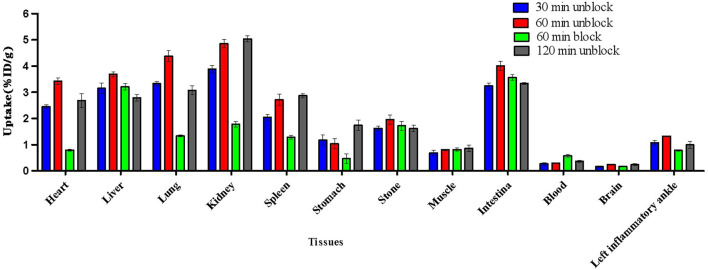
Biodistribution results for ^18^F-VUIIS1008 on day 3 after CFA injection. Data are presented as %ID/g at various times post-injection of ^18^F-VUIIS1008.

**FIGURE 6:**
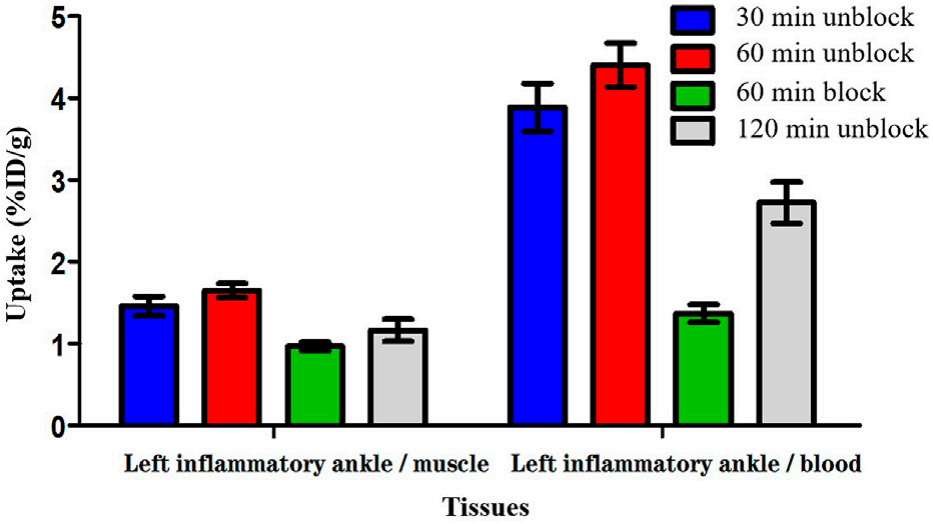
The radioactivity ratios of the left inflammatory ankle to blood (LIA/B) and the left inflammatory ankle to muscle (LIA/M) at various times post-injection based on biodistribution results of ^18^F-VUIIS1008.

The biodistribution studies were conducted on day 3 after CFA injection. At 30, 60, and 120 min post-injection, the biodistribution characteristics of ^18^F-VUIIS1008 was shown in Figure 5. ^18^F-VUIIS1008 displayed high radioactivity uptake in the left inflammatory ankle. At 30, 60, and 120 min, the left inflammatory ankle uptake was 1.08% ± 0.08% ID/g, 1.33% ± 0.02% ID/g, 0.99% ± 0.1 3% ID/g, respectively, lower than that in the liver, kidney, intestine, stomach, lungs, bone, and spleen, whereas it was higher than blood, muscle, and brain. Furthermore, ^18^F-VUIIS1008 showed high levels of the left inflammatory ankle to muscle (LIA/M) and left inflammatory ankle to blood (LIA/B) (Figure 6). At 60 min, the ratio of LIA/M and LIA/B was 1.65 ± 0.07 and 4.40 ± 0.22, respectively, and higher than that at 30 and 120 min.

In order to investigate the specificity of ^18^F-VUIIS1008, an excess of PK11195 (500 μg) was coinjected with ^18^F-VUIIS1008 into RA rats to saturate endogenous and overexpressed TSPO in some normal tissues. PK11195 decreased significantly the accumulations of ^18^F-VUIIS1008 in the left inflammatory ankle and many tissues, such as liver, lung, heart, kidney, stomach, and intestine (*p* <.05), whereas it did not decreased those in the blood, muscle, and bone (*p* >.05).

### 3.5 Longitudinal PET/CT imaging studies

**FIGURE 7:**
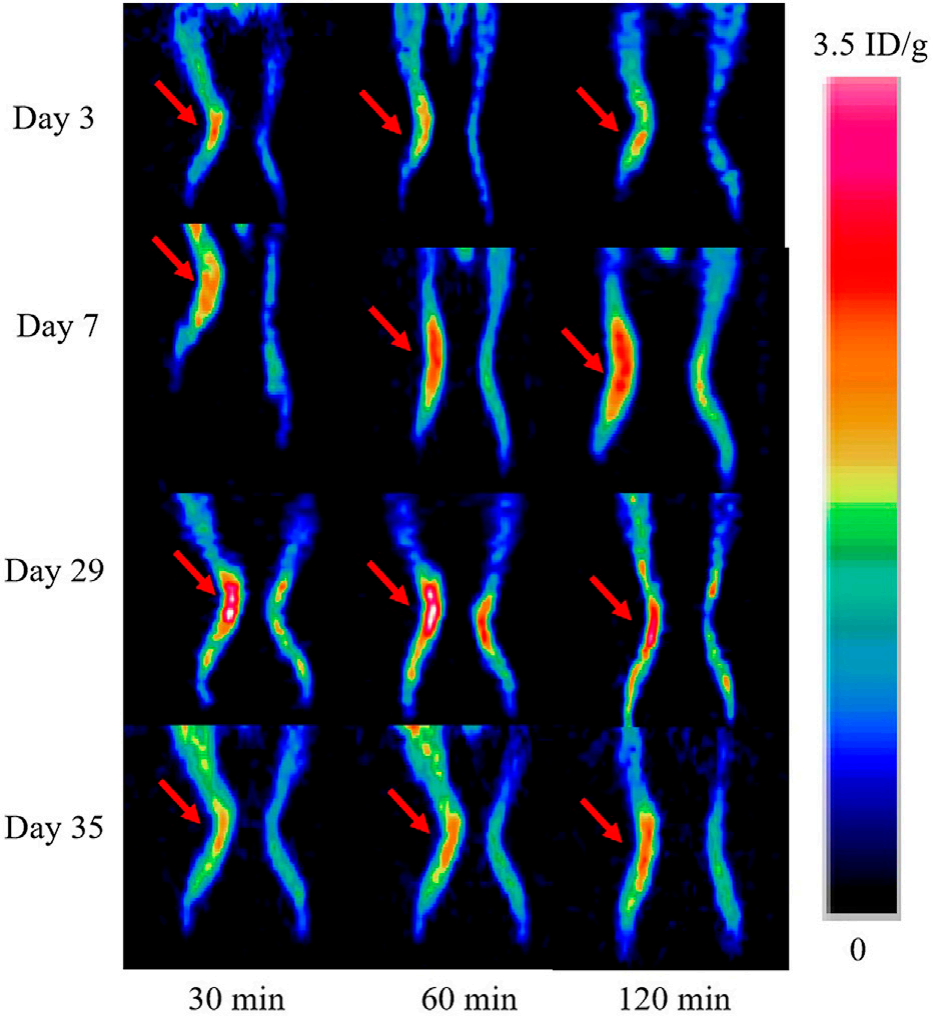
^18^F-VUIIS1008 PET imaging of RA rat model at 30, 60, and 120 min post-injection on day 3, 7, 29, 35 after CFA injection. Red arrow indicate left inflammatory ankles.

**FIGURE 8:**
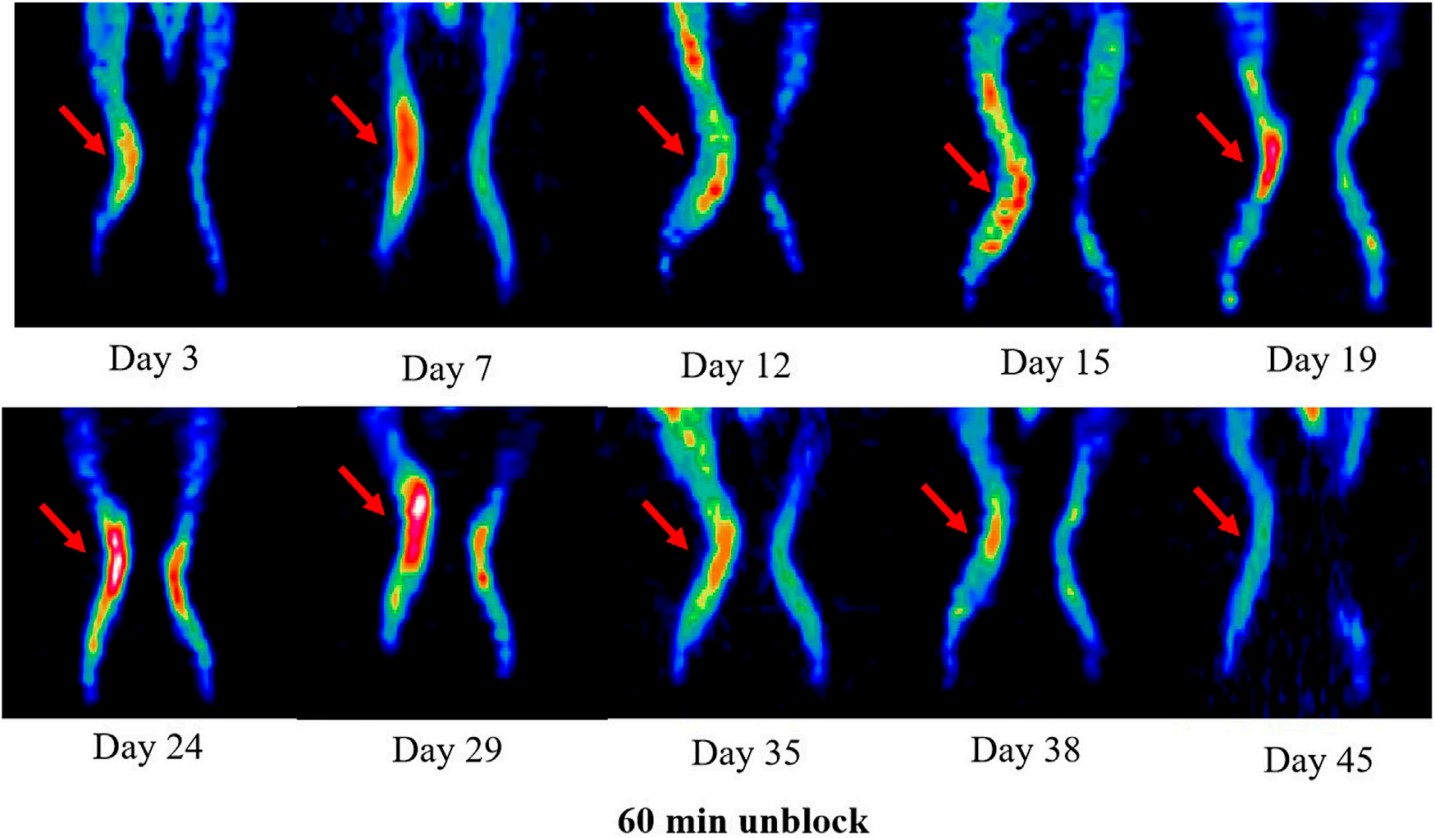
^18^F-VUIIS1008 PET imaging of RA rat model at 60 min post-injection on day 3, 7, 12, 15, 19, 24, 29, 35, 38, and 45 after CFA injection. Red arrow indicate left inflammatory ankles.

**FIGURE 9:**
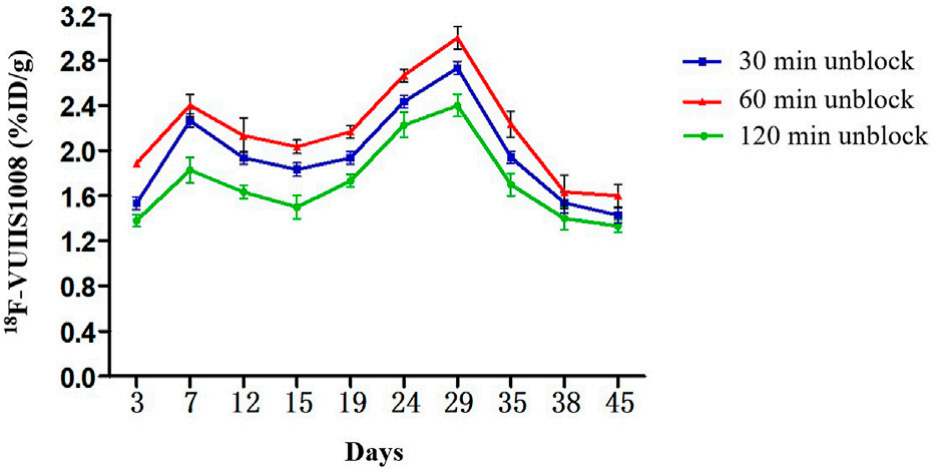
Quantitative analysis of ^18^F-VUIIS1008 uptake in the left inflammatory ankles at different time post-injection on different day based on PET imaging. Peak uptake was found on day 29.

**FIGURE 10:**
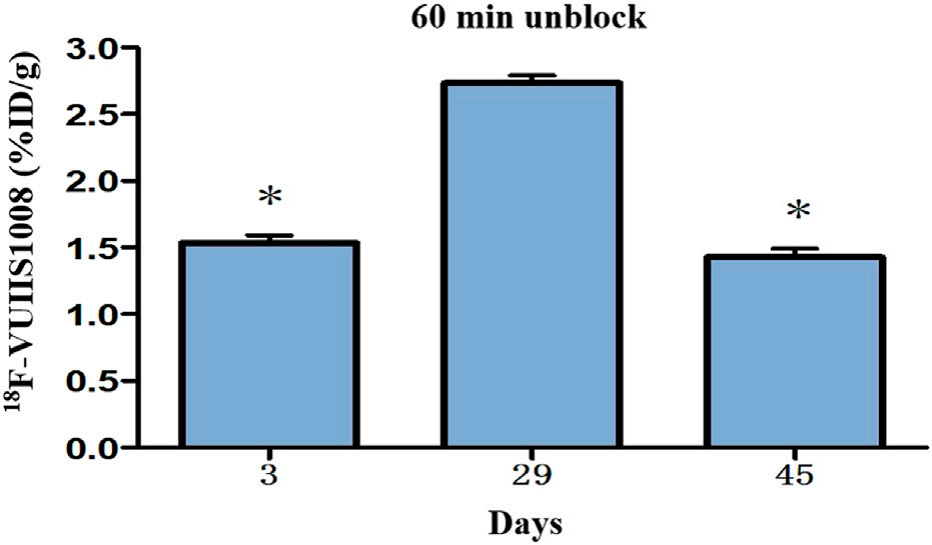
Quantitative analysis of ^18^F-VUIIS1008 uptake in the left inflammatory ankles on day 3, 29, 45 based on PET imaging.

**FIGURE 11:**
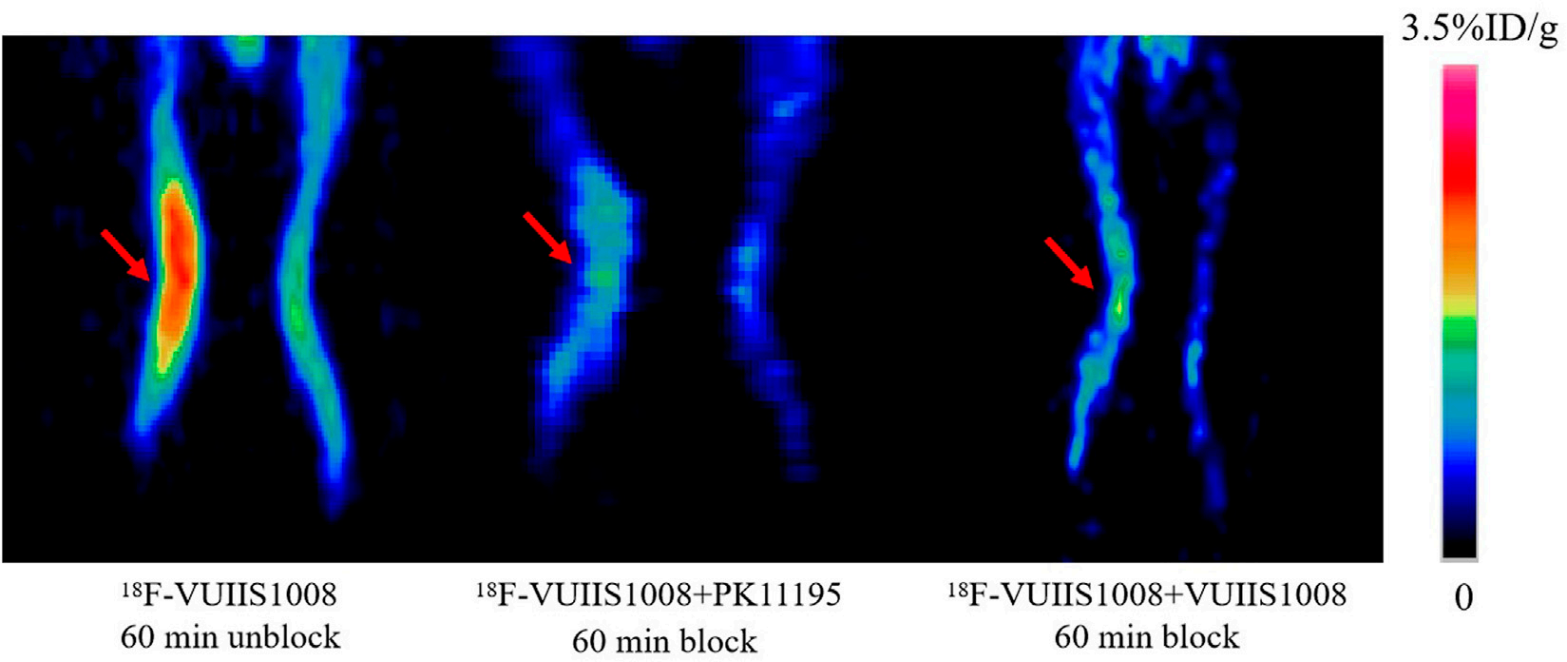
Representative PET images of ^18^F-VUIIS1008 uptake in the left inflammatory ankles without and with cold PK11195 or VUIIS1008 blocking. Red arrow indicate left inflammatory ankles.

**FIGURE 12:**
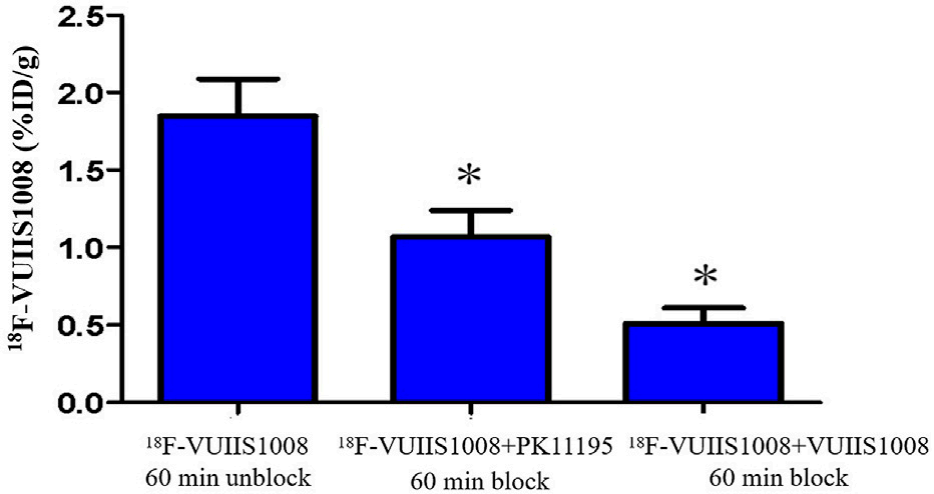
Quantification of ^18^F-VUIIS1008 uptake in the left inflammatory ankles without and with cold PK11195 or VUIIS1008 blocking.

Longitudinal small animal PET/CT studies were performed at 30, 60, and 120 min after injection of ^18^F-VUIIS1008 on day 3, 7, 12, 15, 19, 24, 29, 35, 38, and 45 after CFA injection. As shown in Figure 7, ^18^F-VUIIS1008 highly accumulated in the left inflammatory ankles at 30 min compared with the collateral ankles, and exhibited a gradual increasing uptake during 60–120 min post-injection. The left inflammatory ankles were clearly visible with good inflammatory to background contrast. During day 3–45 after CFA injection, the uptake of left inflammatory ankles was a wiggle trace with two peaks on day 7 and 29, and then the uptake on day 29 was the highest (60 min (3.00% ± 0.08% ID/g) (*p* <.05) (Figures 8–10). Importantly, there was an inflection point on day 15, and after day 15, the uptake gradually increased along with time till day 29, then dropped slowly along with time till day 45, when the uptake was the lowest, and still higher than that in collateral muscle. Furthermore, when co-injected with unlabeled PK11195 (500 μg) or VUIIS1008 (500 μg), the left inflammatory ankles were barely visible on PET images at 60 min post-injection (Figure 11), while the contralateral normal muscle stayed at the low uptake level, affected slightly by PK11195 or VUIIS1008 injection. Regions of interest (ROIs) analysis of PET showed a high ratio of the left inflammatory ankle in RA rats injected unblocking dose compared to with 500 μg blocking dose at 60 min post-injection (Figure 12) (*p* <.05).

### 3.6 Histological results

**FIGURE 13:**
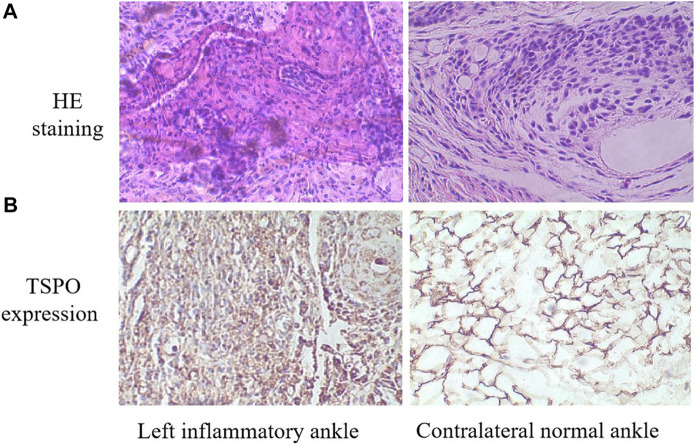
**(A)** HE staining of the left inflammatory ankles and contralateral normal ankles (×100). **(B)** The expression of TSPO on the left inflammatory ankles and contralateral normal ankles by immunohistochemical analysis (×100).

**FIGURE 14:**
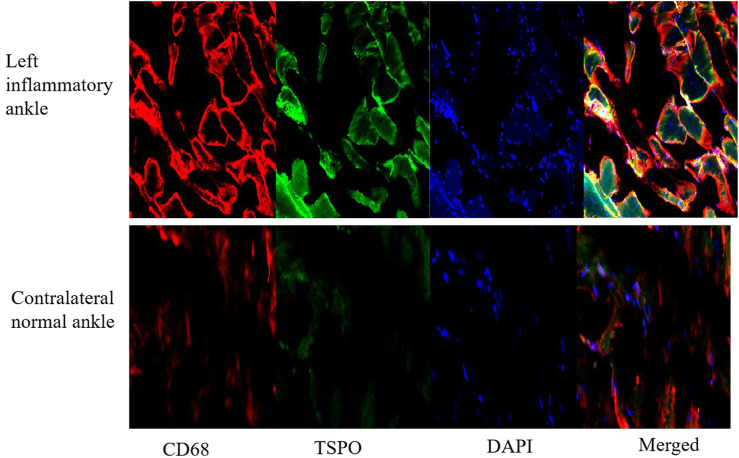
The expression of TSPO and CD68 on contralateral normal ankles by immunofluorescence staining (×100).

For HE tests, it found that synovial hyperplasia and infiltration of inflammatory cells (such as lymphocaytes and macrophages) were identified in the left inflammatory ankles, while they were not observed in the normal contralateral ankles (Figure 13A). For immunohistochemistry (IHC) analysis, there found positive staining of TSPO in the left inflammatory ankles, while negative expression of TSPO in the normal contralateral normal ankles (Figure 13B). Moreover, for immunofluore-scence analysis, it showed the positive staining of TSPO and macrophage (CD68) could be detected in the left inflammatory ankles, whereas they were not found in the normal contralateral normal ankles (Figure 14).

## 4 Conclusion

In this study, we performed longitudinal ^18^F-VUIIS1008 PET imaging to defined the temporal profile of macrophage infiltration in synovitis in rat models of rheumatoid arthritis. The results supported the feasibility of ^18^F-VUIIS1008 PET imaging to identify the dynamics of macrophage activation and infiltration in different stages of synovitis in RA rat models, suggesting ^18^F-VUIIS1008 PET imaging could be used to be a non-invasive imaging technique for clinical management of RA.

## Data Availability

The original contributions presented in the study are included in the article/Supplementary Materials, further inquiries can be directed to the corresponding author.

## Abbreviations

RA, rheumatoid arthritis; TSPO, translocator protein, 18 kDa; PET, positron emission tomography; SPECT, single-photon emission computed tomography; VUIIS1008, 2-(5,7-diethyl-2-(4-(2-flfluoroethoxy)phenyl)pyrazolo[1,5-a]pyrimidin-3 -yl)-N,N-di- ethylacetamide); 18F, fluorine-18; CFA, Complete Freund’s Adjuvant; DMSO, dimethyl sulfoxide; DMEM, Dulbecco’s modifified Eagle’s medium; RAW264.7, mouse macrophage cell lines; PBS, phosphate-buffered saline; LIA/M, the ratio of the left inflflammatory ankle to muscle; LIA/B, the ratio of the left inflammatory ankle to blood; ROI, regions of interest; %ID/g, percentage of the injected radioactivity per gram of tissue.

## References

[B1] Arthur J. S. Ley S. C. (2013). Mitogen-activated protein kinases in innate immunity. Nat. Rev. Immunol. 13 (9), 679–692. 10.1038/nri3495 23954936

[B2] Barrera P. van der Laken C. J. Boerman O. C. Oyen W. J. van de Ven M. T. van Lent P. L. (2000). Radiolabelled interleukin‐1 receptor antagonist for detection of synovitis in patients with rheumatoid arthritis. Rheumatolog- (Oxford) 39 (8), 870–874. 10.1093/rheumatology/39.8.870 10952741

[B3] Calabrò A. Caterino A. L. Elefante E. Valentini V. Vitale A. Talarico R. (2016). One year in review 2016: Novelties in the treatment of rheumatoid arthritis.novelties in the treatment of rheumatoid arthritis. Clin. Exp. Rheumatol. 34 (3), 357–372. 27268779

[B4] Capaccione K. M. Doubrovin M. Bhatt N. Mintz A. Molotkov A. (2020). Granzyme B PET imaging of the innate immune response. Molecules 25 (13), 3102. 10.3390/molecules25133102 32646038 PMC7411671

[B5] De Cata A. D'Agruma L. Tarquini R. Mazzoccoli G. (2014). Rheumatoid arthritis and the biological clock. Expert Rev. Clin. Immunol. 10 (5), 687–695. 10.1586/1744666x.2014.899904 24684672

[B6] Firestein G. S. (2003). Evolving concepts of rheumatoid arthritis. Nature 423 (6937), 356–361. 10.1038/nature01661 12748655

[B7] Gatliff J. CampanellaTSPO M. (2016). Tspo: Kaleidoscopic 18-kDa amid biochemical pharmacology, control and targeting of mitochondria. Biochem. J. 473 (2), 107–121. 10.1042/bj20150899 26733718

[B8] Gent Y. Y. Ahmadi N. Voskuyl A. E. Hoetjes N. van Kuijk C. Britsemmer K. (2014). Detection of subclinical syno- vitis with macrophage targeting and positron emission tomography in patients with rheumatoid arthritis without clinical arthritis. J. Rheumatol. 41 (11), 21 45–2152. 10.3899/jrheum.140059 25274888

[B9] Gent Y. Y. Weijers K. Molthoff C. F. Windhorst A. D. Huisman M. C. Kassiou M. (2014). Promising potential of new genera- tion translocator protein tracers providing enhanced contrast of arthritis imag- ing by positron emission tomography in a rat model of arthritis. Arthritis Res. Ther. 16 (2), R70. 10.1186/ar4509 24625077 PMC4060541

[B10] Jalil S. F. Arshad M. Bhatti A. Ahmad J. Akbar F. Ali S. (2016). Rheumatoid arthritis: What have we learned about the causing factors? Pak J. Pharm. Sci. 29 (2), 629–645. 27087104

[B11] Kanegawa N. Collste K. Forsberg A. Schain M. Arakawa R. Jucaite A. (2016). *In vivo* evidence of a functional association between immune cells in blood and brain in healthy human subjects. Brain Behav. Immun. 54, 149–157. 10.1016/j.bbi.2016.01.019 26820224

[B12] Kurowska-Stolarska M. Alivernini S. (2022). Synovial tissue macrophages in joint homeostasis, rheumatoid arthritis and disease remission. Nat. Rev. Rheumatol. 18 (7), 384–397. 10.1038/s41584-022-00790-8 35672464

[B13] Kwon Y. D. Kang S. Park H. Cheong I. K. Chang K. A. Lee S. Y. (2018). Novel potential pyrazolopyrimidine based translocator protein ligands for the evaluation of neuroinflammation with PET. Eur. J. Med. Chem. 159, 292–306. 10.1016/j.ejmech.2018.09.069 30296688

[B14] Liu P. Wang T. Yang R. Dong W. Wang Q. Guo Z. (2020). Preclinical evaluation of a novel 99mTc-labeled CB86 for rheumatoid arthritis imaging. ACS Omega 5 (49), 31657–31664. 10.1021/acsomega.0c04066 33344817 PMC7745438

[B15] Malviya G. Anzola K. L. Podestà E. Laganà B. Del Mastro C. Dierckx R. A. (2012). (99m)Tc-labeled rituximab for imaging B lymphocyte infiltration in inflammatory autoimmune disease patients. Mol. Imaging Biol. 14 (5), 637–646. 10.1007/s11307-011-0527-x 22127469 PMC3443359

[B16] Papadopoulos V. Baraldi M. Guilarte T. R. Knudsen T. B. Lacapère J. J. Lindemann P. (2006). Translocator prot- ein (18kDa): New nomenclature for the peripheral-type benzodiazepine receptor based on its structure and molecular function. Trends Pharmacol. Sci. 27 (8), 402–409. 10.1016/j.tips.2006.06.005 16822554

[B17] Soler Palacios B. Estrada-Capetillo L. Izquierdo E. Criado G. Nieto C. Municio C. (2015). Macrophages from the synovium of active rheumatoid arthritis exhibit an activin A-dependent pro-inflammatory profile. J. Pathol. 235 (3), 515–526. 10.1002/path.4466 25319955

[B18] Su X. Cheng K. Liu Y. Hu X. Meng S. Cheng Z. (2015). PET imaging of insulin-like growth factor type 1 receptor expression with a 64Cu-labeled Affibody molecule. Amino Acids 47 (7), 1409–1419. 10.1007/s00726-015-1975-4 25854877

[B19] Tang D. McKinley E. T. Hight M. R. Uddin M. I. Harp J. M. Fu A. (2013). Synthesis and structure-activity relationships of 5, 6, 7-subst- ituted pyrazolopyrimidines: Discovery of a novel TSPO PET ligand for cancer imaging. J. Med. Chem. 56 (8), 3429–3433. 10.1021/jm4001874 23521048 PMC3648642

[B20] Tang D. Nickels M. L. Tantawy M. N. Buck J. R. Manning H. C. (2014). Preclinical imaging evaluation of novel TSPO-PET ligand 2-(5, 7-Diethyl-2-(4-(2-[(18)F]fluoroethoxy) phenyl)pyrazolo[1, 5-a]pyrimidin-3-yl)-N, N-diethylacetamide ([ (18)F]VUIIS1008) in glioma. Mol. Imaging Biol. 16 (6), 813–820. 10.1007/s11307-014-0743-2 24845529 PMC4372299

[B21] Tran L. Huitema A. D. van Rijswijk M. H. Dinant H. J. Baars J. W. Beijnen J. H. (2011). CD20 antigen imaging with ^124^I-rituximab PET/CT in patients with rheumatoid arthritis. Hum. Antibodies 20 (1-2), 29–35. 10.3233/hab-2011-0239 21558621

[B22] Udalova I. A. Mantovani A. Feldmann M. (2016). Macrophage heterogeneity in the con- text of rheumatoid arthritis. Nat. Rev. Rheumatol. 12 (8), 472–485. 10.1038/nrrheum.2016.91 27383913

